# Prognostic impact of CD133 expression in Endometrial Cancer Patients

**DOI:** 10.1038/s41598-017-08048-0

**Published:** 2017-08-09

**Authors:** G. Mancebo, J. M. Sole-Sedeno, O. Pino, E. Miralpeix, S. Mojal, L. Garrigos, B. Lloveras, P. Navarro, J. Gibert, M. Lorenzo, I. Aran, R. Carreras, F. Alameda

**Affiliations:** 1 Obstetrics and Gynaecology Department of Hospital Universitari del Mar, Passeig Marítim, 25-29, 08003 Barcelona, Catalonia Spain; 2 Oncology Department of Hospital Universitari de la Vall d’Hebron, Passeig de la Vall d’Hebron, 119-129, 08035 Barcelona, Catalonia Spain; 3 Pathology Department of Hospital Universitari del Mar., Passeig Marítim, 25-29, 08003 Barcelona, Catalonia Spain; 4 Universitat Autónoma de Barcelona, Plaza Cívica, Campus de la UAB, 08193 Bellaterra, Catalonia Spain; 5 Universitat Pompeu Fabra, Doctor Aiguader, 80, 08003 Barcelona, Catalonia Spain; 6 Department of Statistics, IMIM-Hospital del Mar Medical Research Institute, Doctor Aiguader, 88, 08003 Barcelona, Catalonia Spain; 7 Cancer Research Programme, IMIM-Hospital del Mar Medical Research Institute, Doctor Aiguader, 88, 08003 Barcelona, Catalonia Spain

## Abstract

To assess the impact of CD133 expression on the prognosis of endometrioid endometrial carcinoma (EEC). We retrospectively assessed CD133 expression in tissue microarray of 116 surgically treated FIGO I-III EEC. Tumors with ≥10% of CD133-expressing cells were considered CD133-positive (CD133+). On the basis of CD133 expression, clinical and pathological parameters, progression-free survival (PFS) and overall survival (OS) were evaluated. Of the EEC studied 85.2% showed CD133-expressing cells. Only 61% (n = 66) of EEC presented ≥10% of CD133 expressing cells and were considered CD133+. The mean OS for CD133+ tumour patients was 161 months (95% CI, 154–168) as compared with 146 months (95% CI, 123–160) for those with CD133- tumors (p = 0.012). The mean PFS for CD133+ tumour was 159 months (95% CI, 149–168) as compared with 147 months (95% CI, 132-161) in those with a CD133-tumour (p = 0.014). CD133+ tumours were less likely to have vascular invasion (p = 0.010) and more likely to be well differentiated (p = 0.034). C133+ tumours predicted favorable OS and PFS of EEC patients, with a Hazard Ratio 4.731 (95% CI, 1.251–17.89; p = 0.022). CD133+ tumor status correlates with favorable prognosis of EEC. Our findings are in agreement with studies addressing brain and colorectal tumours.

## Introduction

Endometrial cancer is the most common gynaecological malignancy worldwide. Of the two clinic pathological variants recognized, almost 80% of cases are endometrioid or Type I^1^. Although more than 75% of patients with Type 1 endometrial cancers are diagnosed at an early stage with uterine-confined disease, approximately 15% of these cancers will recur and the patients will die as a result of the disease^2^. Identifying patients at increased risk of relapse has become a priority after the diagnosis of endometrial cancer. These patients are likely to benefit from more extensive surgery, adjuvant treatment or targeted therapies that improve prognosis. Risk factors associated with disease relapse include age, tumour grade, and lymphatic, vascular and myometrial invasion^3^. However, ancillary diagnosis with immunohistochemical markers would better reflect the biological behaviour of tumours and the molecular mechanisms underlying progression and relapse.

Of particular interest are cancer stem cells (CSCs). The theory of CSCs argues that only a small subpopulation of tumour cells has the capacity to initiate and maintain tumour growth. CSCs have stem cell-like properties such as self-renewal, unlimited proliferative potential, and the capability to differentiate into multiple cell types. Because of these properties, CSCs are referred to as tumour-initiating cells. Moreover, recent data provide evidence to support the hypothesis that CSCs are also responsible for cancer recurrence and resistance to treatment, thus they condition prognosis^4–10^.

Different markers like CD133, CD44, and CD117 are expressed on the surface of CSCs^11, 12^. CD133 is a five-transmembrane glycoprotein identified as a useful cell surface marker for the detection of tumour-initiating cells. Previous studies have reported the involvement of CD133 in several solid tumour types, including colorectal, brain, prostate, and lung^7, 13–17^. To date, few studies have provided evidence of CD133+ endometrial CSCs^6, 18–20^.

To our knowledge, little attention has been devoted to the relationship between CD133 tumour status and prognosis in endometrioid endometrial cancer (EEC) patients^20^.

The aim of this study was to identify different subtypes of EEC based on the percentage of CD133-expressing cells, to evaluate the impact of CD133 expression on survival of EEC patients and to analyse the correlation of CD133 tumour status with relevant clinic-pathological features.

## Materials and Methods

### Patients And Specimens

This retrospective study included patients who had surgically treated FIGO (International Federation of Gynaecology and Obstetrics) stage I to III EEC and available follow-up information in any database of NHS (National Health Service) from July 1999 and December 2008. Clinical and pathological data such as patient’s age, FIGO stage, depth of myometrium invasion, lymphatic vessels involvement, vascular infiltration, and lymph node metastasis were obtained from the medical records. The duration of follow-up was defined as the time between diagnoses and disease recurrence (Progression-free survival, PFS), death (Overall survival, OS), or last medical appointment to avoid differences in follow-up in patients with less aggressive disease.

### Ethics Approval

The study was approved by the Ethics Committee of the Hospital Universitari del Mar number *2014/5837* and was carried out in compliance with the guidelines of the Declaration of Helsinki, Fortaleza, Brazil, 2013. A waiver of informed consent was obtained from the Institutional Ethical Review Board from the home Institution.

### Immunohistochemical Analysis

We assessed CD133 immunohistochemical staining (IHC) in a tissue microarray (TMA) of 116 primary human endometrial cancer specimens.

First, a histological review was performed by a pathologist on haematoxylin and eosin (HE) to confirm pathological diagnostic and to identify areas of tumour mass and the invasive edge. For TMA construction, four fields from each slide were used, one representative slide per case, two of them from the tumour and two more from the invasive front. TMA was performed with a 1-mm needle using Microarrayer (Chemicon, California, USA).

IHC staining for CD133 was performed as follows: the primary antibody (CD133/1 (AC133) pure, human clone W6b3c1, MACS, Milteny Biotech, Cologne, Germany) was applied following the manufacturer’s instructions. Briefly, the retrieval antigen was done with citrate buffer pH6 in an autoclave. The slides were then rinsed with PBS and incubated with the primary antibody at 1/100 dilution overnight at 4 °C. The slides were rinsed again with PBS and incubated with the secondary antibody for 1 hour at room temperature. The slides were revealed with DAB. H was used as counterstain. After processing all the samples, we considered only 108 cases, because of technical problems with the IHC staining for 8 samples.

**Figure 1:**
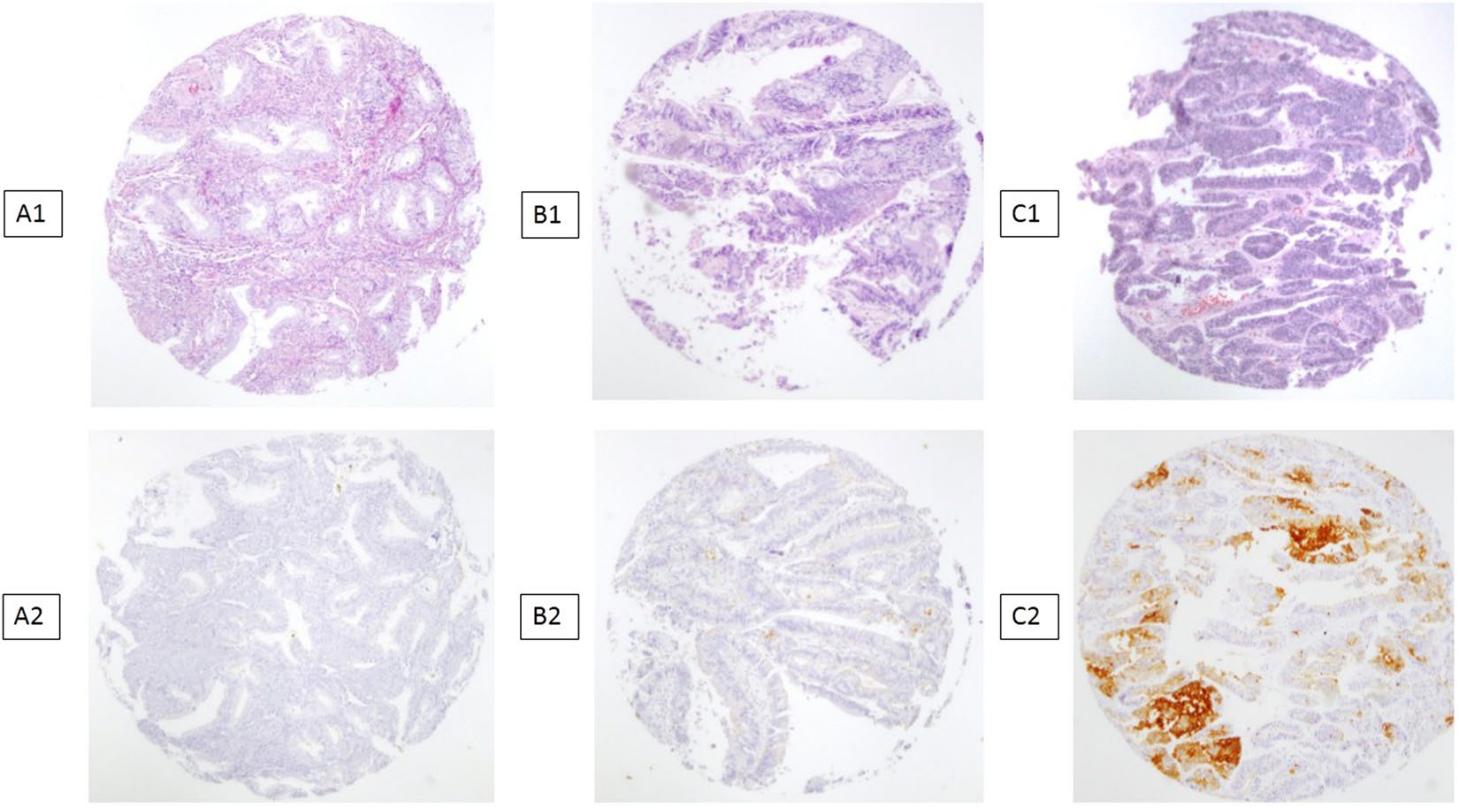
Endometrioid adenocarcinoma of the endometrium. A1 B1 C1: HE*10. Glandular neoplastic endometrioid tumor. A2: CD133− immunostaining. endometrioid endometrial carcinoma grade 1. B2: CD133+ immunostaining <10% endometrioid endometrial carcinoma grade 1. C2: CD133+ immunostaining >10% endometrioid endometrial carcinoma grade 1.

We defined positive staining as luminal surface expression of CD133 (Fig. 1) either in the middle of the tumour or at the invasive edge. At least the 50% of the surface of the cell was required to be positive to count the overall cell as positive. For practical purposes, CD133 positivity was defined on the basis of the percentage of positive cells in cancer-affected areas. For this purpose, for each patient, we took into account the mean number of CD133-expressing cells in the two samples of tumour and in the two samples from the invasive edge. Tumours with CD133 expression in over 10% of whole tumour area were considered CD133+. Two pathologists (FA, OP) were required for the evaluation of slides, and discordance was resolved by consensus.

### Statistical Analysis

The X^2^ test was used to analyse the association between tumour status for CD133 and categorical data of relevant clinical and pathological features.

To study PFS and OS, Kaplan-Meier survival plots were generated based on CD133 status. Curves were compared using the log-rank test, to assess statistical significance Cox proportional hazards regression model was used to analyse the independent prognostic factors, showing Hazard Ratio (HR) and 95% confidence interval (CI). A p value of <0.05 was considered to be statistically significant.

All statistical analyses were carried out using the SPSS software package version 18.0 (SPSS, Chicago, IL).

## Results

In this study, 116 cases of surgically treated Endometrioid Endometrial Carcinoma FIGO Stage I to III were identified. The characteristics of the patients are summarized in Table 1. The mean age of patients was 66 years (range: 45–83) and mean follow-up 82.43 months (range 2–171).

**Table 1:** Patient and Tumor Characteristics (n: 116).

	Condition, n(%)		
Menopause	No 27 (23.8%)	Yes (89, 76.2%)	
Parity	No (19, 19.6%)	1: (21, 21.6%)	>1: 57, (58.7%)
EEC Grade	1 79 (68.1%)	2: 24 (20.7%)	3: 13 (11.2%)
FIGO Stage	I (96 (82.8%)	II: 11 (9.5%)	III: 9 (7.8%)
Myometrial infiltration	No: 5 (4.3%)	<50%: 53 (45.7%)	>50%: 57 (49.1%)
Vascular invasion	No: 103 (88.8%)	Yes: 13 (11.2%)	
Peritumoral inflammation	No: 34 (29.6%)	Yes: 81 (70.4%)	
Leucocytes infiltration	No: 75 (65.2%)	Yes: 40 (34.8%)	

A total of 108 patients with EEC were finally included in the CD133 analysis because of technical problems with the IHC staining. We assessed the percentage of CD133 expression of the whole tumour area of the 108 samples available. The TMA revealed 85.2% of EEC samples showing CD133-expressing cells. The relative percentage of CD133-expressing cells ranged from 0% to 58%. CD133 was detected in a median of 13% tumour cells per patient. 61% of EECs presented CD133 expression in over 10% of whole tumour area and were consequently considered CD133+ tumours.

**Figure 2:**
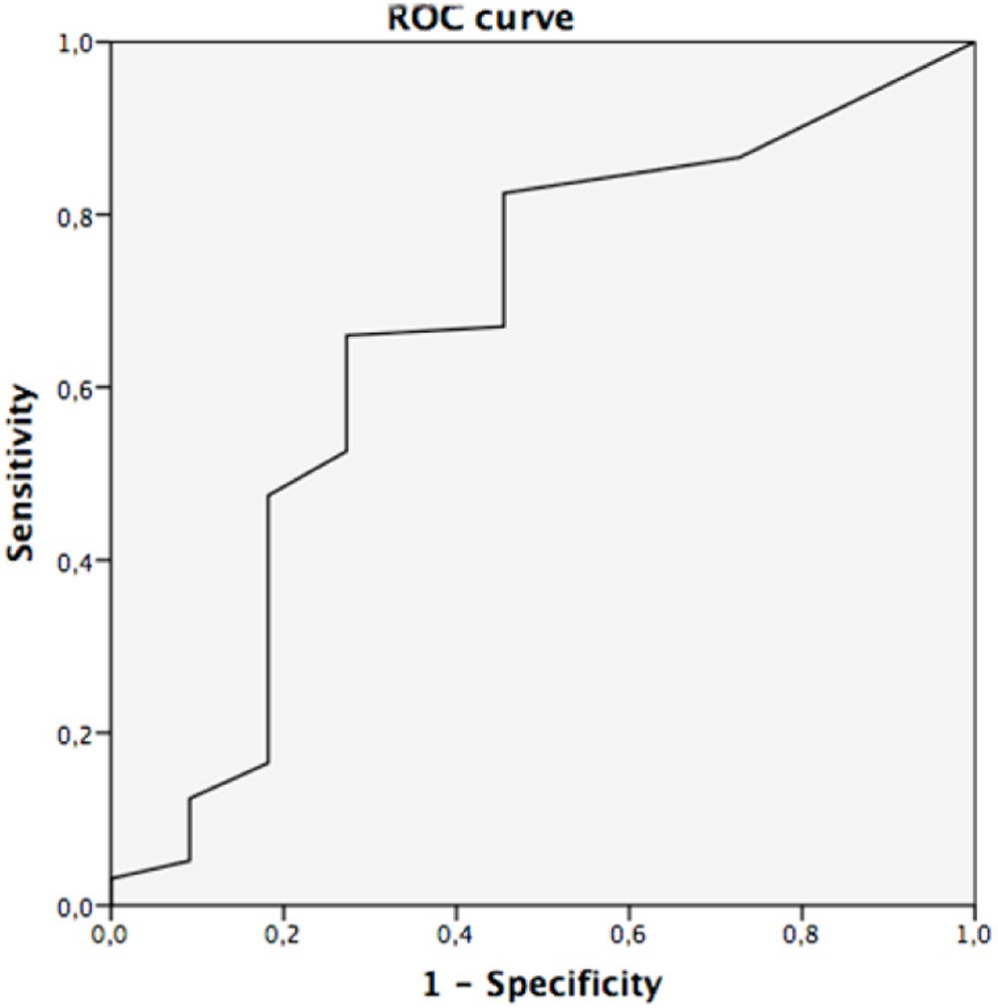
ROC curve *for determining CD133 cut off value*.

A mild correlation (Rho Spearman: 0.216, p = 0.013) was found between survival time and CD133% expression. A ROC curve was constructed to determine the sensitivity and specificity of CD133 expression to predict survival at 5 years (Fig. 2). The Area Under the Curve was 0.667. The best cut-off point for CD133 expression was 10% with a sensitivity of 64.6% and a specificity of 73%.

CD133 status and its relation with clinical and pathological parameters are shown in Table 2. There was no significant relationship between CD133+ tumours status and FIGO stage, myometrial invasion, lymphatic vessels infiltration or presence of peritumoural infiltrating lymphocytes (p > 0.05). However CD133+ status differed depending on the histological grade (p = 0.034) and degree of vascular invasion (p = 0.010). In this regard, CD133+ tumours were less likely to have vascular invasion and were more likely to be well differentiated, regardless of their extent.

**Table 2:** CD133 Tumor Status and pathological parameters (n: 108).

	CD 133 Tumor Status		P
	**Negative**	Positive	
**Figo State**			
**1**	33	56	0.32
**2**	6	4	
**3**	3	6	
**Graded**			
1	23	49	0.034
2	11	12	
3	8	5	
**Myometrial INFI**			
NO	1	3	0.053
<50%	17	35	
>50%	23	28	
**Vasular Invasion**			
NO	33	63	0.010
YES	9	3	
**Inflamation**			
NO	13	18	0.161
YES	29	47	
**Leucocites Presence**			
NO	26	43	0.148
YES	16	22	

**Figure 3:**
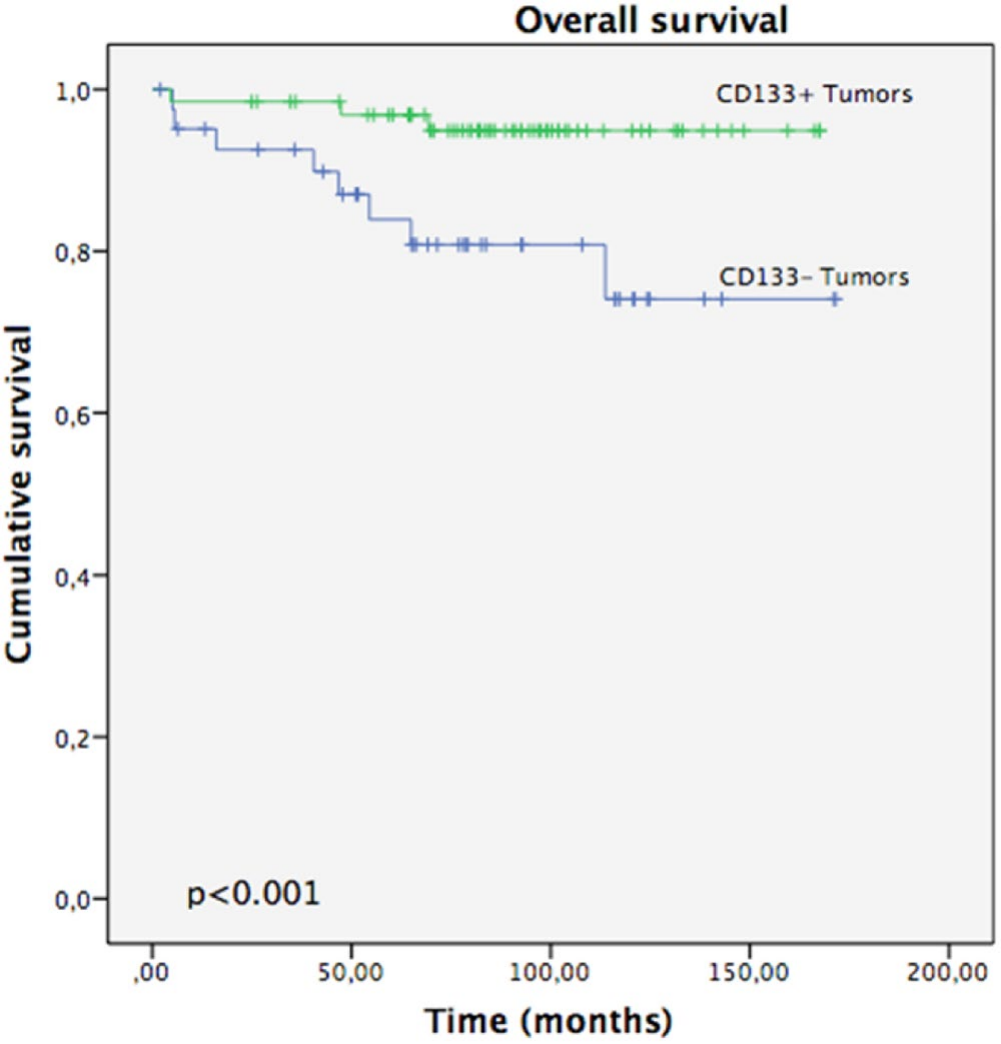
Overall Survival: CD133+ vs CD133− Tumors.

**Figure 4:**
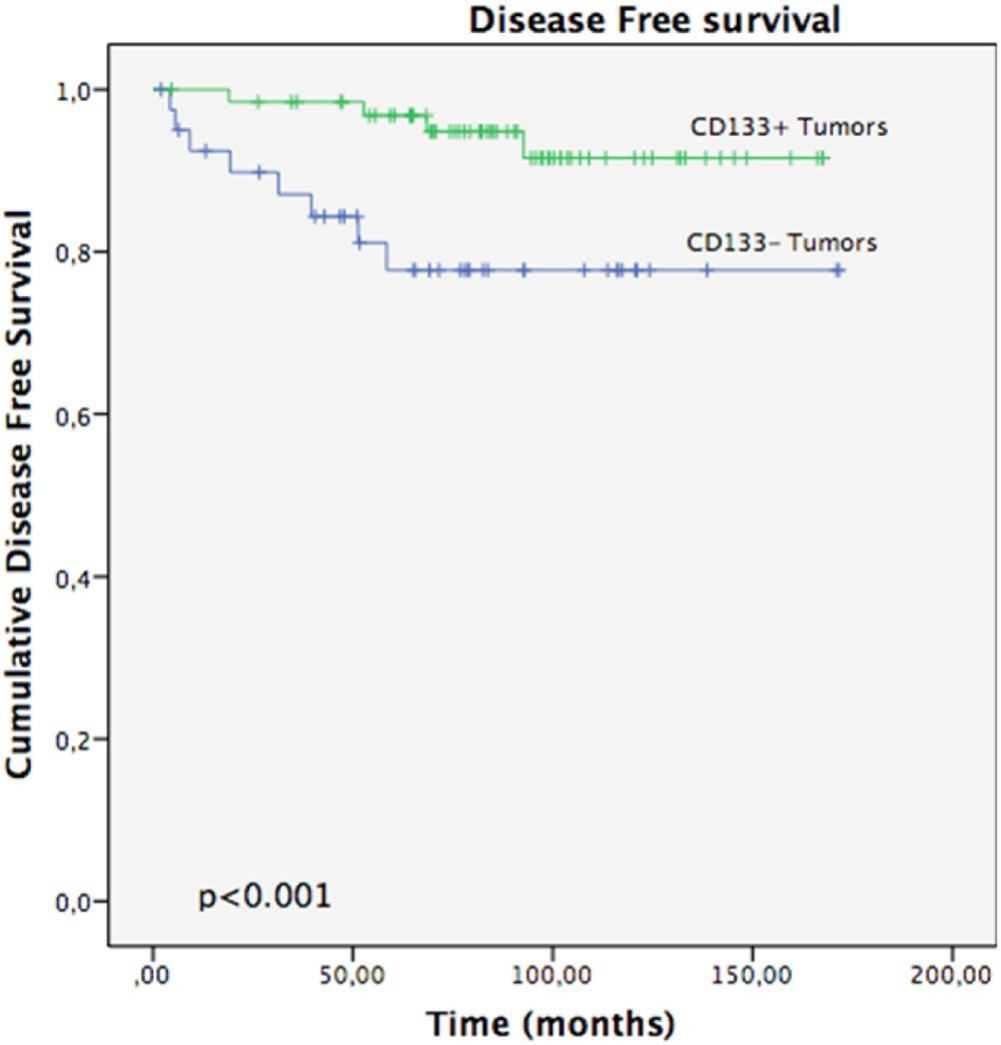
Progression Free Survival: CD133+ vs CD133− Tumors.

After 5 years of follow-up, 89.8% of the EEC patients were still alive, 83.6% of them with no evidence of disease. We assessed Overall Survival (OS) on the basis of tumour status for CD133 (Fig. 3). For patients with CD133+ tumours, mean OS was 161 months (95% CI, 154–168) compared with 141 months (95% CI, 123–160) for those with CD133-ones (p = 0.012). We also evaluated the effect of CD133 tumour status on Progression Free Survival (PFS) (Fig. 4). At the time of the last follow-up, 9.5% of patients had relapsed. The mean PFS was also significantly better in those with CD133+ tumours, with a mean of 158 month (95% CI, 150–167) vs. 139 months (95% CI, 120–159) in those CD133−tumours (p = 0.014).

Univariate and multivariate Cox proportional hazards regression analyses showed that CD133+ tumours predicted better OS and PFS of EEC patients (HR of 4.731 (95% CI, 1.251–17.89), p = 0.022).

## Discussion

To the best of our knowledge, there are very few studies that study the utility of CD133+ tumour status as a prognostic factor in primary untreated endometrial cancer.

The theory of CSCs argues that only a small subpopulation of tumour cells has the capacity to initiate and maintain tumour growth. It is also believed that these cells are responsible for recurrence and resistance to treatment^9, 15, 22, 23^.

Although normal stem cells have been identified in normal endometrial tissue^24^, to date only the CD133 marker has been proposed for the identification and isolation of endometrial CSCs^6, 7, 24^.

Using flow cytometric analysis, Friel *et al*.^7^ reported that the percentage of CD133+ cells in primary human endometrioid endometrial carcinoma samples ranged from 5.7% to 27.4% and also showed that CD133+ cells had greater tumour-initiating capacity relative to their CD133− counterparts. In the same way, Rutella *et al*.^6^ described a variable degree of CD133 immunoreactivity, reporting a median of 18.1% of CD133 expressing cells (range, 1.3–62.6%). Besides, similar to what reported by Nakamura^21^, they showed that CD133+ cells have greater proliferative potential and a higher response to estradiol, and were less sensitive to paclitaxel and cisplatin than the CD133- population. All these findings are consistent with CSCs properties, thus supporting the role of CD133 as a marker of endometrial CSCs. Similarly, in our patient cohort, CD133 expression was detected in a median of 13% of tumours (range, 0–58%).

According to the literature^1^, 82.8% of cases in our cohort were EEC FIGO 1 and 68.1% were grade 1, despite 7.3% of FIGO 1 cases recurred. A key challenge in EEC is to accurately predict the risk of recurrence and to identify high-risk patients between this that a priori has a good prognosis. Such identification would allow clinicians to provide them more appropriate treatments with more extensive surgery and adjuvant therapy. We found no correlation between CD133+ tumour status and clinical features. Of special interest was the observation of a relationship between CD133+ status, grade, and vascular invasion. CD133+ tumours were less likely to have vascular infiltration and poor histological differentiation. On the basis of these findings, we propose that tumour status for CD133 would be considered a prognostic factor, regardless of the extent of disease at the time of diagnosis, as it correlates better with the histological features present in the tumour.

Although our data didn’t consider percentages of CD133 expressing cells and do differentiate between CD133− and CD133+ tumours, they support the results reported by Rutella *et al*.^6^. Rutella *et al*. found higher percentages of CD133-expressing cells in patients with early-endometrioid tumours compared with those in advanced stages of disease. Moreover, they observed a higher expression of CD133 in patients with no detectable lymph node metastases. However, they did not find a statistically significant correlation between histological grade and percentage of tumour cell expression of CD133. This may have been because of the low number of grade 1 samples included in their study. Rutella et al. proposed that a subpopulation of CD133−expressing cells would have a greater potential to limit disease extension and recurrence. Along with the same line, several studies on colorectal cancer^25^ and glioblastomas^26^ revealed that the down-regulation of CD133 expression is associated with the acquisition of metastatic potential and more aggressive behaviour. This down-regulation of CD133+ cells may result in the emergence of a CD133− population with greater aggressiveness and progression. However, the biological plausibility of these hypotheses is yet unproven.

Finally, in order to establish the prognostic value of CD133 tumour status in primary EEC, we attempted to correlate the expression of CD133 with clinical outcome. Our findings were somewhat unexpected, although in line with the above affirmations. CSCs are supposed to play a role in cancer relapse and resistance to treatment and to be predictive of poor clinical outcome and lower patient survival^9, 22, 23^. Correlations between the proportion of CSCs and poor clinical outcome have been described for glioma^27, 28^, colon^29^, breast^30^ and prostate^31^ tumours.

However, our data do not support the findings of earlier research^21^. In our series, patients with CD133+ tumours showed longer OS and PFS than CD133-tumours. Moreover, according to our findings, CD133+ tumours have greater predictive capacity for OS and PFS of EEC patients than CD133-tumours, showing a Hazard Ratio of 4.7 (p = 0.022). These observations indicate that signalling pathways other than CD133 may be involved in the CSC-like properties. The findings of Rutella support this notion^6^. They showed that both CD133+ and CD133− cells have the ability to induce tumour growth in nude mice, thereby supporting the idea that not all tumour-initiating cells express CD133. Our data are consistent with those published by others who failed to demonstrate CD133 as an independent prognostic marker for predicting poor outcome in some cases of colorectal and brain cancer^29–31^. However, our data disagree with those obtained by Nakamura *et al*.^21^. They examined the prognostic value of CD133 immunohistochemical expression in 62 endometrioid endometrial carcinoma samples and revealed that tumours with high CD133 expression (>1% of expressing cells) showed worse OS than those with weak or absent expression (p = 0.023). This apparent discordance can be explained by the definition of tumours showing high CD133 expression. Nakamura et al. considered high expression as tumours with more than 1% of CD133+ cells. We analysed separately tumours with over 10% of CD133+ cells and those with less than 10%. While CD133+ tumours accounted for 61% of our cohort, tumours showing high CD133 expression (>10%) in the Nakamura’s accounted for only 21%. Moreover, in addition to the apparent discordance in OS and PFS results, the overall prognosis was worse in Nakamura’s patient cohort than in ours. They reported 17.7% of relapses and 19.9% of deaths compared with the 9.5% and 12.9% of deaths respectively in our cohort. These results could be attributed to a lower proportion of cases with high expression (>10%) in Nakamura’s study, which may conceal the possible role of CD133+ tumours as a prognostic factor of favourable outcome.

We would like to point out the potential limitations of our study. One of the most important was the relatively small sample size from only one retrospective study. Also because of retrospective analysis we were unable to evaluate the effect on prognostic of adjuvant treatments in CD 133+ tumours, because of a great heterogeneity of given treatments in these cases. Other concerns are related to the method used to detect CD133 and the criteria for scoring CD133 positivity. As CD133 is not homogenously expressed, we prepared a TMA to fully represent the whole tumour. We chose to detect CD133 by IHC. Although semi-quantitative, IHC staining is a well-established technique widely available in most pathology laboratories, thus facilitating the translation of findings to current clinical practice. Given that different studies use different tissue samples (cell lines vs. human tissue), tissue sampling methods (tissue microarray vs. slides), and criteria regarding the pattern and distribution of CD133 staining hinders comparison. Moreover, CD133 is a glycoprotein that can be detected by monoclonal antibodies recognizing specific glycosylation-dependent epitopes, in particular AC133 (named as CD133/1) and AC141 (named as CD133/2)^32, 33^. Here, we have used an antibody specific for the AC133 antigen, one of the most frequently used in previous studies to identify CSCs in endometrial cancer^6, 21^. Importantly, discordant expression of AC133 and AC141 epitopes have been reported in other tumors^34^ and therefore we cannot rule out that staining of our TMA with CD133/2 could lead to different data. However, despite this limitation, similar detection of CD133 expression was found between our study and those previously reported^6, 7^. On the other hand, different antibodies used can also explain the discordance of the prognostic value of CD133 between our data and those by Nakamura *et al*.^21^ because a polyclonal antibody for CD133 was used in their immunohistochemistries while we used the CD133/1 monoclonal antibody.

On the basis of our results, we propose that CD133 tumour status is a useful tool for the management of EEC. CD133− status at the time of therapeutic planning, can improve the identification of patients with a priori good prognosis but with high-risk tumours that would require extended lymphatic surgery or selectively targeted treatments and longer follow-up.

## Conclusion

The results of the present study show that CD133+ tumour status is correlated with favourable prognosis of EEC patients, independently of the extent of disease at diagnosis. CD133 tumour status emerges as a useful biomarker of low risk EEC. CD133 could be viewed as a complementary tool in the planning of primary treatment for EEC as it provides a more accurate assessment of prognosis and adjuvant treatment. Despite reported data, well-designed prospective and larger studies are needed to confirm our findings. In order to translate findings to clinical practice, it is a priority to standardize methods of detection and criteria for scoring the positivity of CD133. At the time this paper was performed, criteria for CD133 positivity in EEC had still not been established.
